# The complete chloroplast genome of *Eremurus robustus* (Asphodelaceae)

**DOI:** 10.1080/23802359.2019.1674198

**Published:** 2019-10-04

**Authors:** Dilmurod Makhmudjanov, Ziyoviddin Yusupov, Davlatali Abdullaev, Tao Deng, Komiljon Tojibaev, Hang Sun

**Affiliations:** a University of Chinese Academy of Sciences, Beijing, China;; b Key Laboratory for Plant Diversity and Biogeography of East Asia, Kunming Institute of Botany, Chinese Academy of Sciences, Kunming, China;; c National Herbarium of Uzbekistan, Institute of Botany, Academy Sciences of Uzbekistan, Tashkent, Uzbekistan

**Keywords:** Chloroplast genome, phylogenetic analysis, *Eremurus robustus*

## Abstract

*Eremurus robustus* Regel (*Eremurus,* Asphodelaceae) is an ornamental plant and endemic to the Tien Shan and Pamir Alai mountains. In this study, the complete chloroplast genome of *E. robustus* was determined. The cp genome of *E. robustus* is 155,647 bp in length consisting of a large single-copy (LSC) region of 84,776 bp, a small single-copy (SSC) region of 17,786 bp, and a pair of identical inverted repeat regions (IRs) of 26,490 bp. The cp genome encodes 114 unique genes, including 80 protein-coding genes, 4 ribosomal RNAs and 30 transfer RNAs. Among them, ten coding genes contained one intron each and two genes (*clpP* and *ycf3*) contained two introns each. The phylogenetic analysis demonstrates a close relationship between genus *Aloe* with genus *Eremurus.*

The genus *Eremurus* M.Bieb is naturally distributed in Central and West Asia, extending East to China and West to Turkey and Ukraine (Xinqi et al. 2000). It can be easily distinguished from other genera of Asphodelaceae by having flowers more than 50, leafless flowering stem and rhizomatous rootstock (Fedchenko 1935) *Eremurus robustus* Regel is an ornamental plant endemic to the Tien Shan and Pamir Alai mountains. The roots of *E. robustus* are used for glue extraction and food. Here, we characterise the complete cp genome of *E. robustus* in an effort to provide genomic and genetic sources useful for further research on ornamental important species of the family Asphodelaceae.

Fresh leaves of *E. robustus* were collected from Uzbekistan, Kashkadarya province, Langar village (E66° 40′ 580″, N39° 24′ 990″). Voucher specimen of *E. robustus* was deposited in the National Herbarium of Uzbekistan (TASH, DM0012), Institute of Botany, Uzbekistan Academy of Sciences. Total DNA was extracted according to the modified CTAB method (Doyle and Doyle, 1987), then fragmented into 200 bp for library construction and sequenced using an Illumina HiSeq 2500 system at BGI (Shenzhen, Guangdong, China). We assembled the cp genome based on the methods of Jin et al. (2018), and the plastome of *Aloe maculata* was used as a reference genome (Genbank accession: NC_035505). We annotated the cp genome of *E. robustus* using Geneious v11.1.5 (Kearse et al. 2012), then start and stop codons and intron/exon boundaries were manual edited. Phylogenetic analysis was performed using RAxML-HPC BlackBox v8.1.24 software (Stamatakis 2006) with the GTRGAMMAI model. The cp genome sequence of *E. robustus* was submitted to GenBank with the accession number of MN315570.

The cp genome of *E. robustus* was 155,647 bp in length, containing a large single copy region (LSC) of 84,881 bp (GC-35.1%), a small single copy region (SSC) of 17,786 bp (GC-31.1%), and a pair of inverted repeats (IR) regions of 26,490 bp (GC-42.8%). A total of 114 genes were identified including 80 protein-coding genes, 30 tRNA genes, four ribosomal RNA genes. Among these, twelve coding genes contained introns. The phylogenetic analysis of 16 chloroplast genomes showed that *E*. *robustus* is closely clustered with *A. maculata* (Figure 1). The cp genome sequence of *E. robustus* will be useful for further population genomic studies, phylogenetic analyses, and genetic engineering studies of family Asphodelaceae.

**Figure 1.**
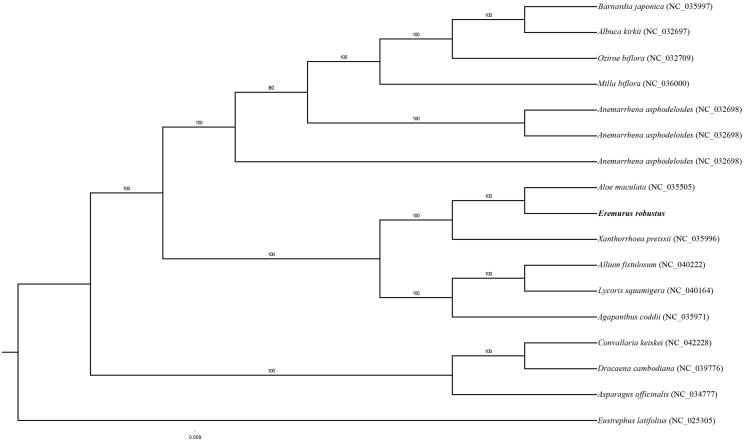
Phylogenetic analysis of E. robustus with 16 related species. Numbers in the nodes are the bootstrap values from 1000.
